# Sonographic features of carotid artery dissection due to extension of aortic dissection: a case report

**DOI:** 10.1186/s13089-019-0147-2

**Published:** 2019-12-02

**Authors:** Christian Boßelmann, Sven Poli

**Affiliations:** 1 Department of Neurology and Epileptology, and Hertie-Institute for Clinical Brain Research, Eberhard-Karls University of Tübingen, Hoppe-Seyler-Straße 3, 72076 Tübingen, Germany; 2 Department of Neurology and Stroke, and Hertie-Institute for Clinical Brain Research, Eberhard-Karls University of Tübingen, Hoppe-Seyler-Straße 3, 72076 Tübingen, Germany

**Keywords:** Neurology, Ultrasound, Point-of-care systems, Carotid artery dissection

## Abstract

**Background:**

Carotid artery dissection due to extension of aortic dissection (CAEAD) is a severe complication of acute aortic dissection. The risk of ischemic stroke is increased. Early sonographic detection and repeat evaluation are necessary to guide clinical management.

**Case presentation:**

A 58-year-old male patient presents with sudden, tearing retrosternal pain. Point-of-care carotid ultrasound is used to establish the diagnosis of CAEAD. We describe a number of sonographic features and compare ultrasound to other imaging modalities.

**Conclusions:**

Bedside carotid ultrasound enables rapid, sensitive and safe hemodynamic assessment, especially in critically ill patients.

## Background

Aortic dissection (AD) is a clinical emergency. A tear in the tunica intima, the innermost layer of the vessel, is propagated by blood flowing into the false lumen. This dissection membrane can further extend into the aortic branches, including the carotid arteries. This is referred to as carotid artery extension of aortic dissection (CAEAD). Cerebral malperfusion can result from the dissection membrane restricting or occluding blood flow, akin to a carotid stenosis or large-vessel occlusion. Alternatively, artery-to-artery embolization or progressive stenosis from mural thrombosis may occur. Hence, rapid diagnosis and repeat hemodynamic evaluation are necessary to guide clinical management [1].

## Case presentation

**Fig. 1:**
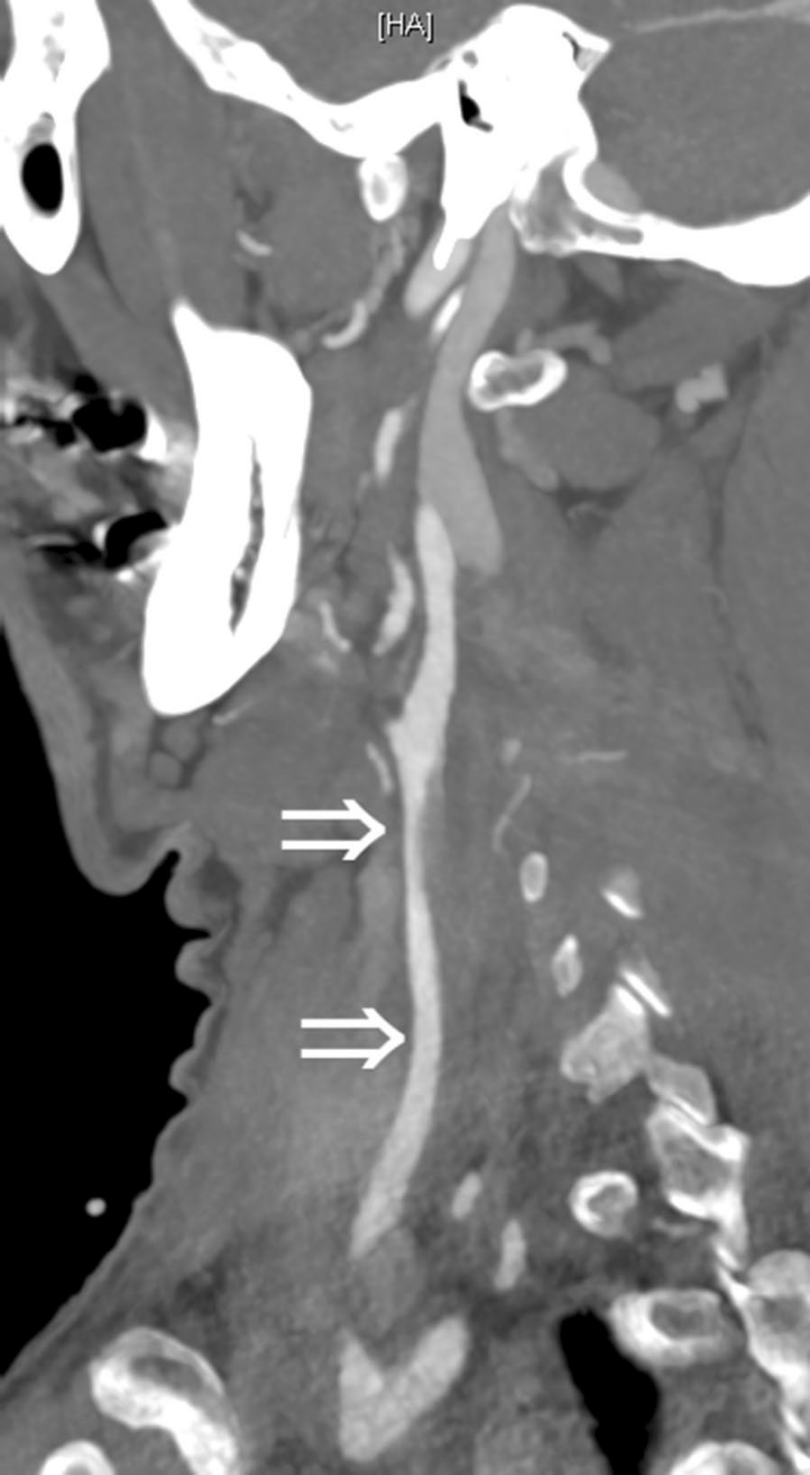
Computed tomography angiography. On sagittal oblique maximum intensity projection (MIP), an extended and smoothly tapering stenosis of the right common carotid artery is seen (arrows)

A 58-year-old male patient presented with sudden tearing retrosternal pain and dyspnea. No traumatic injury was reported. Chest computed tomography angiography (CTA) displayed an acute aortic dissection (Stanford type A, DeBakey type I), extending from the aortic sinus to both common iliac arteries and all supra-aortic branches (Fig. 1). The patient underwent emergency supracoronary ascending aorta replacement and aortic valve repair.

**Fig. 2:**
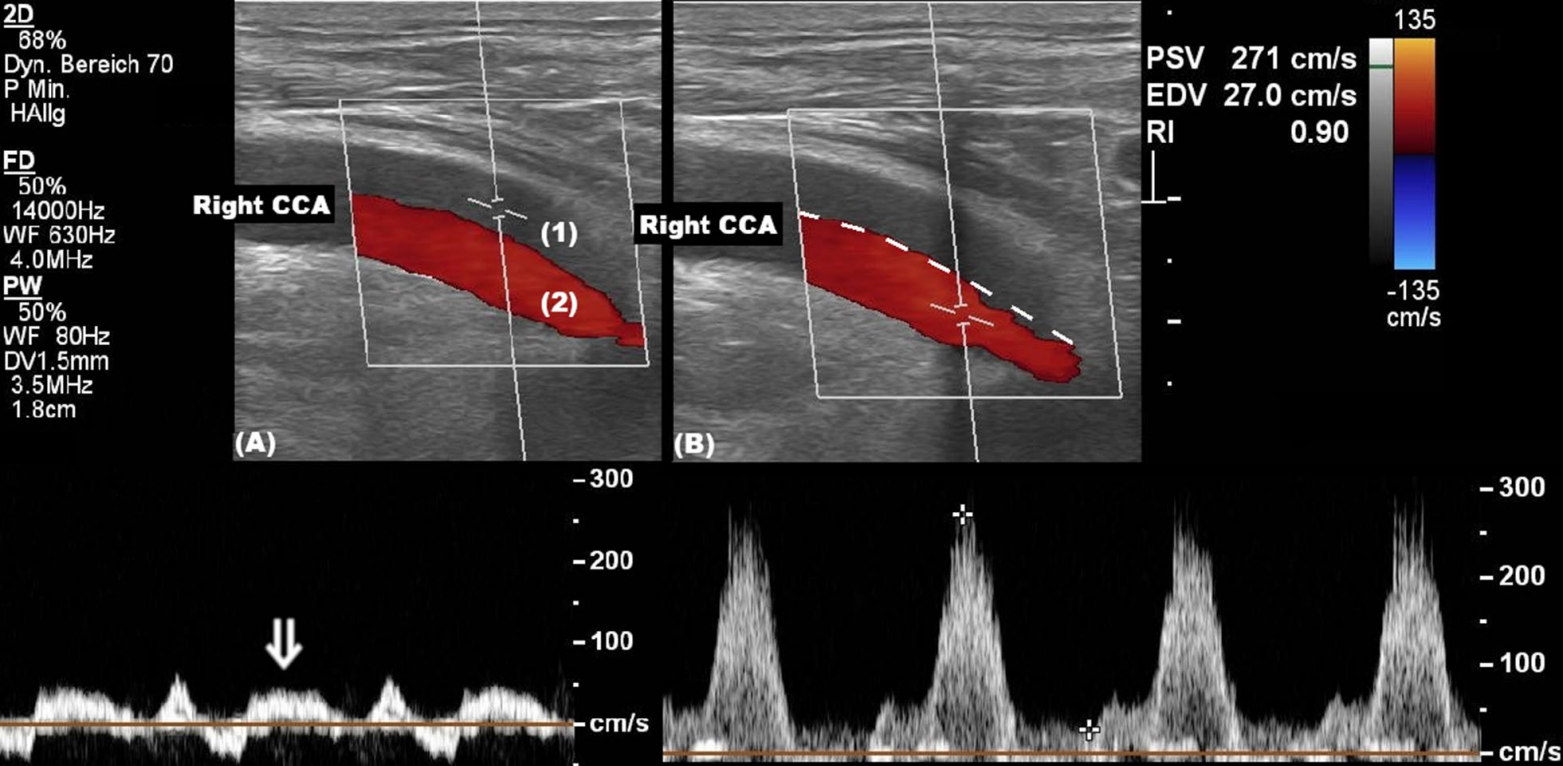
Duplex sonography of the right common carotid artery. Note the dissection membrane (dashed line) separating two distinct lumina, each with different flow profiles. The false lumen (1) exhibits systolic and diastolic orthograde flow (arrow). The true lumen (2) also displays orthograde flow, but is narrowed to a luminal stenosis of 50% (peak systolic velocity 271 cm/s, reference range 29–178 cm/s [2])

Postoperatively, dynamic evaluation through pulsed-wave mode Doppler (8 MHz, vascular probe) and brightness-mode (B-mode) ultrasound demonstrated several sonographic features typical of a dissection. Notably, a dissection membrane was seen extending from the proximal right common carotid artery (CCA) to the distal ICA (Fig. 2, Additional file 1: Video S1). Flow direction in the true lumen was orthograde. No collateral flow was noted on transcranial Doppler, i.e., both the ophthalmic artery and the anterior cerebral artery were found to have normal, orthograde flow. Thus, distal high-grade stenosis or occlusion was excluded.

The left carotid arteries were also examined. On B-mode ultrasound, no dissection membrane or any of the features described above were observed. However, Doppler ultrasound demonstrated a high peak systolic flow velocity of 470 cm/s in the proximal left CCA (reference range 29–178 cm/s [2]), indirectly suggesting bilateral CCA dissection (not pictured).

## Discussion

Here, we report a case where bedside carotid ultrasound enabled early detection and hemodynamic assessment of CAEAD in a critically ill patient. A number of sonographic features are shown, such as luminal stenosis, a perfused false lumen, and a dissection membrane. Features not present include a thickened and hypoechogenic vessel wall, intracranial stenosis or occlusion, and pseudoaneurysm [3].

Four imaging modalities are primarily used in the diagnosis of carotid dissections. Digital subtraction angiography (DSA), i.e., contrast-enhanced invasive vascular imaging, remains the gold standard. Dynamic magnetic resonance angiography (MRA) may also be performed, especially in patients with iodinated contrast allergy or renal impairment. Fat-saturation sequences are useful to detect intramural hematoma, even if the lumen itself is not narrowed. Compared to DSA, MRA offers a variable performance at a sensitivity of 50–100% and a specificity of 29–100% [4] and is considered by the American Heart Association and the American Stroke Association to be the best initial screening test [5]. However, the speed of image acquisition is slow and MRA is not always feasible in intensive care unit (ICU) patients. Conversely, CTA is widely available, offers rapid image acquisition and compares favorably with DSA at a sensitivity of 64–100% and specificity of 67–100% [6].

Carotid ultrasound offers a number of advantages over static vessel imaging. Doppler mode yields information on flow characteristics including the peak systolic as well as diastolic flow velocities. This method is used to diagnose and grade stenosis and is especially valuable in the indirect assessment of the petrous ICA segment, where CTA is limited due to vessel wall calcifications or skull base artifacts. Using additional brightness-mode (B-mode) imaging with color-coded Doppler significantly increases the sensitivity in detecting carotid dissections, as compared to MRA, with no false-positive diagnoses [7]. Carotid ultrasound is also portable, which enables bedside examination and avoids unnecessary transports in ICU patients, which are associated with a number of serious adverse events [8]. Lastly, carotid ultrasound is the least invasive method, avoiding radiation exposure or the administration of a contrast agent. Hence, bedside carotid ultrasound is a safe and effective imaging modality in carotid dissections, suitable for use in critically ill patients.

Limitations may apply. Minor vessel damage, such as an intimal tear or small mural hematoma, may not be readily apparent on ultrasound. This applies especially to locations where B-mode imaging cannot be carried out, such as the skull base. Here, alternate imaging modalities, such as fat-saturation MRI or (contrast-enhanced) MRA, may be useful as complementary diagnostic tools.

## Conclusion

This case study demonstrates the effective use of bedside carotid ultrasound to diagnose carotid artery dissection due to extension of aortic dissection (CAEAD). This enabled rapid decision-making in regards to further medical management.

## Supplementary information

**Additional file 1: Video S1.** B-mode ultrasound of the right common carotid artery, examined continuously, starting in the proximal segment and moving the transducer distally. (A) Transverse view. (B) Sagittal view. Note the dissection membrane, a linear and mobile hyperechogenic structure, extending from the proximal CCA to the distal ICA, separating the false (1) and true (2) lumina.

## Data Availability

All data generated or analyzed during this study are included in this published article and its additional files.

## Abbreviations

AD: aortic dissection
CAEAD: carotid artery extension of aortic dissections
CCA: common carotid artery
CTA: computed tomography angiography
DSA: digital subtraction angiography
ECA: external carotid artery
ICA: internal carotid artery
ICU: intensive care unit
MRA: magnetic resonance angiography
